# Quadratic Frequency Modulation Signals Parameter Estimation Based on Two-Dimensional Product Modified Parameterized Chirp Rate-Quadratic Chirp Rate Distribution

**DOI:** 10.3390/s18051624

**Published:** 2018-05-19

**Authors:** Zhiyu Qu, Fuxin Qu, Changbo Hou, Fulong Jing

**Affiliations:** College of Information and Communication Engineering, Harbin Engineering University, Harbin 150001, China; quzhiyu@hrbeu.edu.cn (Z.Q.); fuxin@hrbeu.edu.cn (F.Q.); jingfl@hrbeu.edu.cn (F.J.)

**Keywords:** quadratic frequency modulation (QFM) signal, parametric symmetric self-correlation function (PSSAF), nonuniform fast-Fourier transform (NUFFT), fast-Fourier transform (FFT)

## Abstract

In an inverse synthetic aperture radar (ISAR) imaging system for targets with complex motion, the azimuth echo signals of the target are always modeled as multicomponent quadratic frequency modulation (QFM) signals. The chirp rate (CR) and quadratic chirp rate (QCR) estimation of QFM signals is very important to solve the ISAR image defocus problem. For multicomponent QFM (multi-QFM) signals, the conventional QR and QCR estimation algorithms suffer from the cross-term and poor anti-noise ability. This paper proposes a novel estimation algorithm called a two-dimensional product modified parameterized chirp rate-quadratic chirp rate distribution (2D-PMPCRD) for QFM signals parameter estimation. The 2D-PMPCRD employs a multi-scale parametric symmetric self-correlation function and modified nonuniform fast Fourier transform-Fast Fourier transform to transform the signals into the chirp rate-quadratic chirp rate (CR-QCR) domains. It can greatly suppress the cross-terms while strengthening the auto-terms by multiplying different CR-QCR domains with different scale factors. Compared with high order ambiguity function-integrated cubic phase function and modified Lv’s distribution, the simulation results verify that the 2D-PMPCRD acquires higher anti-noise performance and obtains better cross-terms suppression performance for multi-QFM signals with reasonable computation cost.

## 1. Introduction

The high-resolution inverse synthetic aperture radar (ISAR) imaging has been widely used in the field of civil and military in the past few decades. The ISAR image processing and relative algorithms are described in [1,2]. The primary procedures for ISAR imaging algorithm are the range alignment [3,4,5,6] and the phase adjustment after range compression. For targets with smooth motions, conventional range-Doppler (RD) method is effective in reconstructing ISAR images. For targets with the slow maneuvering, the azimuth echo signals are always modeled as the linear frequency modulation (LFM) signal. The RD method can not acquire a high quality image due to the Doppler frequency shift caused by the chirp rate. Therefore, many algorithms for LFM signal parameters estimation have been proposed, such as Wigner–Ville distribution (WVD) [7], fractional Fourier transform (FrFT) [8], cubic phase function (CPF) [9], Lv’s distribution (LVD) [10] and parameterized centroid frequency-chirp rate distribution (PCFCRD) [11]. However, the azimuth echoes of targets with complex motion should be modeled as the multicomponent quadratic frequency modulation (multi-QFM) signals, and aforementioned algorithms can not be applied for the complex maneuvering targets.

Existing QFM signal parameter estimation algorithms can be divided into non-correlation algorithms and correlation algorithms. Compared with correlation algorithms, two classical non-correlation algorithms, the maximum likelihood (ML) method [12] and the quantification-based method [13] have no cross-terms and perform better in low signal-to-noise ratios (SNR). However, due to their huge computations, the non-correlation algorithms are not suitable in real-time applications. Two representative correlation algorithms, the high order ambiguity function (HAF) [14] and cubic phase function (CPF) [9,15,16], reduce the order of the QFM signal by multilinear and bilinear transforms. They are effective for mono-QFM signal under the high SNR situation and suffer from the cross-term problem when multicomponent QFM (multi-QFM) signals are considered. In order to solve the cross-term problem, the product HAF (PHAF) [17] and the product CPF (PCPF) [18,19] are proposed. Compared with HAF and CPF, the PHAF and PCPF suppress the cross-terms and improve the threshold SNR by multiplying the different HAF and CPF, which are related to the delay variable. However, their estimation accuracy can not meet the requirements in low SNR and their cross-term suppression ability is still poor. Igor Djurovic proposed HAF-integrated CPF (HAF-ICPF) [15] by combining HAF with ICPF to estimate the parameters of the QFM signals. It improves the anti-noise performance to some extent and its threshold SNR is −2 dB [15,20]. However, the HAF-ICPF suffers from the error propagation due to the dechirp procedure. In 2015, Yanyan Li proposed the modified Lv’s distribution (MLVD) [21], which transforms the QFM signals into the chirp rate-quadratic chirp rate (CR-QCR) domain to estimate the CR and the QCR. MLVD solves the error propagation. Furthermore, MLVD improves the cross-term suppression ability further and decreases threshold SNR into −4 dB [21]. However, several disadvantages still exist in these two algorithms. Firstly, the anti-noise performance of the HAF-ICPF and MLVD is still poor. Secondly, the MLVD and HAF-ICPF suffer from the identifiability problem for multi-QFM signals with the same QCR.

In this paper, we propose two-dimensional product modified parameterized chirp rate-quadratic chirp rate distribution (2D-PMPCRD) to estimate the parameters of QFM signals. It combines a multi-scale parametric symmetric self-correlation function (PSSAF) with modified nonuniform fast Fourier transform-Fast Fourier transform (mNUFFT-FFT) to transform the signals into the chirp rate-quadratic chirp rate(CR-QCR) domains. By the operation of PSSAF and mNUFFT-FFT, the auto-terms of multi-QFM signals are transformed into different CR-QCR domains at the same position, while the cross-terms are transformed into different positions on CR-QCR domains. By multiplying all the CR-QCR domains, the 2D-PMPCRD can greatly suppress the cross-terms while strengthening the auto-terms. The operation of multiplying all the CR-QCR domains can also improve the anti-noise performance. Therefore, 2D-PMPCRD has a better cross-term suppression performance and a better anti-noise performance. In addition, the 2D-PMPCRD does not need brute-force search, by using FFT and NUFFT. Thus, the 2D-PMPCRD is suitable in the real-time ISAR imaging system.

The reminder of this paper is organized as follows. In Section 2, the QFM signal model and the principle of 2D-PMPCRD are introduced. In this section, the implementation is presented in discrete-time form. In Section 3, the cross-term suppression performance is discussed. The parameter selection criterion is also analyzed. In Section 4, anti-noise performance for mono-QFM signal and multi-QFM signals is analyzed. The computation of 2D-PMPCRD is also analyzed in this section. Finally, conclusions are given in Section 5.

## 2. The 2D-PMPCRD Method

The geometry for ISAR imaging of targets with complex motions used here is based on the model in [22]. The algorithms of the range alignment and the phase adjustment are described in [6,7,8,9,23,24]. In this paper, we mainly study the processing of the Doppler frequency shift. The ISAR usually transmits the LFM signals. According to [25,26], we can find that the azimuth echoes take the multi-QFM signals model:

(1) $$s\left(t\right)=\sum_{i=1}^{P}A_{i}\exp\left\{j2\pi \left(\phi_{1,i}t+\frac{1}{2}\phi_{2,i}t^{2}+\frac{1}{6}\phi_{3,i}t^{3}\right)\right\}+z\left(t\right),$$

where *t* ($-\frac{T_{s}*N}{2}\leq t\leq \frac{T_{s}*N}{2}$) denotes the slow time. *N* and $T_{s}$ denote the signal length and sampling interval. *P* is the number of QFM signal components. $A_{i}$, $\phi_{1,i}$, $\phi_{2,i}$, and $\phi_{3,i}$ denote the amplitude, the centroid frequency, the CR and the QCR of the *i*th QFM signal, respectively. z(t) denotes the white Gaussian noise, which has a mean of zero and a variance of $\delta^{2}$.

### 2.1. The Principle of 2D-PMPCRD

In order to make the algorithm easily understandable, a noise-free QFM signal is presented as

(2) $$s\left(t\right)=A\exp\{j2\pi \left(\phi_{1}t+\frac{1}{2}\phi_{2}t^{2}+\frac{1}{6}\phi_{3}t^{3}\right)\},$$

where *A*, $\phi_{1}$, and $\phi_{2}$, $\phi_{3}$ denote the amplitude, the centroid frequency, the CR and QCR of the mono-QFM signal, respectively.

Based on the analysis in [11,21], a novel PSSAF with multi-scaled factors for QFM signal parameters estimation is proposed. It can be defined as

(3) $$\begin{array}{c} R_{s}\left(t,\tau ,D\right)=s\left[t+D+\left(\tau +\frac{1}{2}h\right)\right]s\left[t+D-\left(\tau +\frac{1}{2}h\right)\right]\\ s^{*}\left[t-D+\left(\tau +\frac{1}{2}h\right)\right]s^{*}\left[t-D-\left(\tau +\frac{1}{2}h\right)\right], \end{array}$$

where ∗ denotes the complex conjugation, and τ is the lag-time variable. $D=\{d_{i}\},1\leq i\leq L$ denotes the set of multi-scale factors. According to [21], the scaled factor $d_{i}$ is introduced to complete the order reduction, which can benefit the anti-noise performance. *h* denotes the constant delay. It is introduced to improve the algorithm performance in low SNR [10,11]. Thus, the selection of $d_{i}$ and *h* is important for the proposed algorithm. The selection criterion of $d_{i}$ and *h* will be discussed in Section 3.2.

A new time-quadratic chirp rate analysis technique can be obtained by performing Fourier transform on $R_{s}\left(t,\tau ,D\right)$ with respect to τ:

(4) $$\begin{array}{c} \mathrm{TQC}\left(t,\omega ,D\right)=\int_{\tau }R_{s}\left(t,\tau ,D\right)\exp\left[-j2\pi \omega {\left(\tau +\frac{1}{2}h\right)}^{2}\right]d\tau . \end{array}$$

By substituting Equation (2) into Equation (4), we can obtain

(5) $$\begin{array}{c} \mathrm{TQC}\left(t,\omega ,D\right)=\int_{\tau }C\left(D\right)\exp\left[j2\pi \left(4\phi_{2}Dt+2\phi_{3}Dt^{2}\right)\right]\\ \exp[-j2\pi \left(\omega -2\phi_{3}D\right){\left(\tau +\frac{1}{2}h\right)}^{2}]d\tau , \end{array}$$

where C(D) denotes

(6) $$C\left(D\right)=A^{4}\exp\left\{j4\pi \left(2\phi_{1}D+\frac{1}{3}\phi_{3}D^{3}\right)\right\}.$$

Equation (5) is similar to the time-chirp rate (TCR) analysis in [11]. Thus, it has a similar characteristic to the TCR technique and it concentrates along $\omega =2\phi_{3}D$. However, TQC will not concentrate along the *t*-axis and it will cause poor cross-terms suppression and poor anti-noise performance. In order to make Equation (5) concentrate along the *t*-axis, the matrix $H\left(t,\omega \right)=\exp\left(-j2\pi \omega t^{2}\right)$ is firstly introduced to dechirp TQC. After the dechirp processing, we perform the Fourier transform for the dechirped TQC with respect to the *t*-axis. The modified parameterized chirp rate-quadratic chirp rate distribution (MPCRD) is obtained:

(7) $$\begin{array}{c} \mathrm{MPCRD}\left(r,\omega ,D\right)=\int_{0}^{+\infty }\mathrm{TQC}\left(t,\omega ,D\right)\exp\left(-j2\pi \omega t^{2}\right)\exp\left(-j2\pi rt\right)dt\\ =G_{p}sinc\left[\frac{T}{2}\left(r-4\phi_{2}D\right)\right]\int_{0}^{+\infty }\exp\left[-j2\pi \left(\omega -2\phi_{3}D\right){\left(\tau +\frac{1}{2}h\right)}^{2}\right]d\tau , \end{array}$$

where $G_{p}$, ω and *r* denote the amplitudes, quadratic chirp rate domain with respect to τ and chirp rate domain with respect to *t*, respectively. From the analysis of Equation (7), it is easy to see that it coherently accumulates at $\left(r,\omega \right)=\left(4\phi_{2}D,2\phi_{3}D\right)$ on chirp rate and quadratic chirp rate (CR-QCR) domains. QFM signal will be transformed into multi-peaks on CR-QCR domains. The peaks are related to the scale factors. We set $d_{1}$ as the reference scale factor, and the related peak as the reference peak. The relationships between peaks can be presented as

(8) $$\begin{array}{c} P_{1,i}=\frac{\omega_{1}}{\omega_{i}}=\frac{r_{1}}{r_{i}}=\frac{d_{1}}{d_{i}},\left(1\leq i\leq L\right), \end{array}$$

where $P_{1,i}$ is the zoom factors, and $\omega_{1}$ and $r_{1}$ are the coordinates of reference peak. $\omega_{i}$ and $r_{i}$ are the coordinates of peaks, which are related to scale factors $d_{i}$. *L* denotes the number of the scale factors. From Equation (8), we can see that the peaks are related to the scale factors, which are selected by selection criteria. The selection criteria will be discussed in Section 3.2. According to the relationships between peaks and scale factors, the 2D-PMPCRD is proposed, which can be presented as

(9) $$\begin{array}{c} Q_{2D-PMPCRD}\left(r,\omega ,D\right)=\prod_{i=1}^{L}MPCRD\left(r,\omega ,d_{i}\right). \end{array}$$

### 2.2. Implementation of the 2D-PMPCRD

The key steps of the proposed algorithm are the Fourier transform with respect to τ and *t*. In this section, we propose mNUFFT-FFT to transform QFM signals into CR-QCR domains. The mNUFFT-FFT perform modified NUFFT along the τ-axis and modified FFT along the *t*-axis to speed up the implementation.

The discrete form of Equation (3) can be expressed as Equation (10) based on the sampling scheme of Classen and Mecklenbrauker [27] (CM):

(10) $$\begin{array}{c} R_{s}\left(n,m,D\right)=s\left[n+D+\left(m+\frac{h}{2T_{s}}\right)\right]s\left[n+D-\left(m+\frac{h}{2T_{s}}\right)\right]\\ s^{*}\left[n-D+\left(m+\frac{h}{2T_{s}}\right)\right]s^{*}\left[n-D-\left(m+\frac{h}{2T_{s}}\right)\right], \end{array}$$

where $T_{s}$ denotes the sampling interval. $n=-\frac{N}{2},-\frac{N}{2}+1,\ldots ,\frac{N-1}{2}$, $m=-\frac{N}{2},-\frac{N}{2}+1,\ldots ,\frac{N-1}{2}$.

By performing the discrete Fourier transform for Equation (10) with respect to *m*, a new time-quadratic chirp rate technique with the optimal scale factor, which is presented as $d_{1}$, can be represented as

(11) $$\begin{array}{c} TQC\left(n,\omega ,d_{1}\right)=\sum_{m}R_{s}\left(n,m,d_{1}\right)\exp\left\{j2\pi \omega {\left(mT_{s}+\frac{h}{2}\right)}^{2}\right\}. \end{array}$$

In Equation (11), the order of the integration variable is two. Thus, FFT can not be used to speed up the implement. However, through the analysis of [11,28,29], we can use NUFFT to speed up the Fourier transform along the *m*-axis without accuracy loss. The processing of the NUFFT can be described as

(12) $$\begin{array}{c} TQC\left(n,\omega ,d_{1}\right)=NUFFT_{m}\left[R_{s}\left(n,m,d_{1}\right)\right], \end{array}$$

where $NUFFT_{m}\left[*\right]$ denotes the NUFFT process with respect to the *m*-axis.

Then, the matrix $H\left(n,\omega \right)=\exp\left(-j2\pi \omega n^{2}\right)$ is used to dechirp TQC. After that, FFT is used to speed up the Fourier transform implement with respect to *n*-axis. The processing can be described as

(13) $$\begin{array}{c} MPCRD\left(r,\omega ,d_{1}\right)=FFT_{n}\left[TQC\left(n,\omega ,d_{1}\right)H\left(n,\omega \right)\right] \end{array}$$

From Equation (9), we can see that 2D-PMPCRD needs to transform the QFM signal into the same position. However, the auto-terms will be transformed into different positions with different scale factors. Thus, this paper proposes mNUFFT-FFT to speed up the implement and transform the auto-terms into the same position.

In the mNUFFT-FFT, in order to transform the QFM signal into the same position along the *m* axis, the NUFFT with respect to *m* axis can be rewritten as follows:

(14) $$\begin{array}{c} TQC\left(n,\omega ,d_{i}\right)=\sum_{m}R_{s}\left(n,m,d_{1}\right)\exp\left\{j2\pi \omega_{\Delta }{\left(mT_{s}+\frac{h}{2}\right)}^{2}\right\}, \end{array}$$

where $\omega_{\Delta }=\omega_{1}/P_{1,i}\left(1\leq i\leq L\right)$ denotes the modified QCR domain. $\omega_{1}$ denotes the reference QCR domain. Equation (14) is called mNUFFT, the auto-terms with different scale factors will be transformed into the same position along the *m*-axis after the mNUFFT. We can use mFFT with respect to the *n*-axis to transform the auto-terms with different scale factors into the same position along the *n*-axis after the dechirp processing. The processing of mFFT can be presented as

(15) $$\begin{array}{c} MPCRD\left(r,\omega ,d_{i}\right)=\sum_{n}TQC\left(n,\omega ,d_{1}\right)\exp\left(-j2\pi \omega n^{2}\right)\exp\left(-j2\pi r_{\Delta }n\right), \end{array}$$

where $r_{\Delta }=r_{1}/P_{1,i}\left(1\leq i\leq L\right)$ denotes the modified CR domain. $r_{1}$ denotes the reference CR domain.

Finally, the discrete 2D-PMPCRD can be obtained

(16) $$\begin{array}{c} Q_{2D-PMPCRD}\left(r,\omega ,D\right)=\prod_{i=1}^{L}MPCRD\left(r,\omega ,d_{i}\right), \end{array}$$

where *L* is the number of scale factors.

## 3. Cross-Term Suppression Performance Analysis

In this section, the cross-term suppression of 2D-PMPCRD and MLVD are analyzed. The parameter selection criterion is also discussed in this section.

### 3.1. Cross-Term Suppression Comparison

In this section, the cross-term suppression for 2D-PMPCRD and MLVD is discussed under multi-QFM signals. In order to explain the cross-term problem, two QFM signals are considered firstly. It can be presented as

(17) $$\begin{array}{c} s_{multi}\left(t\right)=\sum_{i=1}^{2}A_{i}\exp\left\{j2\pi \left(\phi_{1,i}t+\frac{1}{2}\phi_{2,i}t^{2}+\frac{1}{6}\phi_{3,i}t^{3}\right)\right\}+z\left(t\right)\\ =A_{1}\exp\left\{j2\pi \left(\phi_{1,1}t+\frac{1}{2}\phi_{2,1}t^{2}+\frac{1}{6}\phi_{3,1}t^{3}\right)\right\}+\\ A_{2}\exp\left\{j2\pi \left(\phi_{1,2}t+\frac{1}{2}\phi_{2,2}t^{2}+\frac{1}{6}\phi_{3,2}t^{3}\right)\right\}+z\left(t\right). \end{array}$$

In order to make the proposed algorithm more understandable, Equation (3) that denotes PSSAF of 2D-PMPCRD can be rewritten as

(18) $$\begin{array}{c} R_{s}\left(t,\tau ,D\right)=R\left(t+\left(\tau +\frac{h}{2}\right),D\right)R\left(t-\left(\tau +\frac{h}{2}\right),D\right), \end{array}$$

where

(19) $$\begin{array}{c} R\left(t,D\right)=s\left(t+D\right)s^{*}\left(t-D\right). \end{array}$$

Submitting Equations (17) into (19), we can obtain

(20) $$\begin{array}{c} R\left(t,D\right)=R_{auto}\left(t,D\right)+R_{cross}\left(t,D\right), \end{array}$$

where $R_{auto}\left(t,D\right)$ are the auto-terms, $R_{cross}\left(t,D\right)$ are the cross-terms. The proposed algorithm with one scale factor can be described as

(21) $$\begin{array}{c} R\left(t,d_{i}\right)=R_{auto}\left(t,d_{i}\right)+R_{cross}\left(t,d_{i}\right). \end{array}$$

$R_{auto}\left(t,d_{i}\right)$ and $R_{cross}\left(t,d_{i}\right)$ can be presented as

(22) $$\begin{array}{c} R_{auto}\left(t,d_{i}\right)=A_{1}^{2}\exp\left\{j2\pi \left(2\phi_{1,1}d_{i}+\frac{1}{3}\phi_{3,1}d_{i}^{3}\right)\right\}\times \exp\left\{j2\pi \left(2\phi_{2,1}d_{i}t+\phi_{3,1}d_{i}t^{2}\right)\right\}\\ +A_{2}^{2}\exp\left\{j2\pi \left(2\phi_{1,2}d_{i}+\frac{1}{3}\phi_{3,2}d_{i}^{3}\right)\right\}\times \exp\left\{j2\pi \left(2\phi_{2,2}d_{i}t+\phi_{3,2}d_{i}t^{2}\right)\right\}, \end{array}$$

(23) $$\begin{array}{c} R_{cross}\left(t,d_{i}\right)=A_{1}A_{2}\exp\left\{j2\pi \left[\phi_{1,1}d_{i}+\phi_{1,2}d_{i}+\frac{1}{2}\phi_{2,1}d_{i}^{2}-\frac{1}{2}\phi_{2,2}d_{i}^{2}+\frac{1}{6}\phi_{3,1}d_{i}^{3}+\frac{1}{6}\phi_{3,2}d_{i}^{3}\right]\right\}\times \\ \exp\{j2\pi [\left(\phi_{1,1}-\phi_{1,2}+\phi_{2,1}d_{i}+\phi_{2,2}d_{i}+\frac{1}{2}\phi_{3,1}d_{i}^{2}-\frac{1}{2}\phi_{3,2}d_{i}^{2}\right)t+\\ \frac{1}{2}\left(\phi_{2,1}-\phi_{2,2}+\phi_{3,1}d_{i}+\phi_{3,2}d_{i}\right)t^{2}+\frac{1}{6}\left(\phi_{3,1}-\phi_{3,2}\right)t^{3}]\}+\\ A_{1}A_{2}\exp\left\{j2\pi \left[\phi_{1,1}d_{i}+\phi_{1,2}d_{i}+\frac{1}{2}\phi_{2,2}d_{i}^{2}-\frac{1}{2}\phi_{2,1}d_{i}^{2}+\frac{1}{6}\phi_{3,1}d_{i}^{3}+\frac{1}{6}\phi_{3,2}d_{i}^{3}\right]\right\}\times \\ \exp\{j2\pi [\left(\phi_{1,2}-\phi_{1,1}+\phi_{2,1}d_{i}+\phi_{2,2}d_{i}+\frac{1}{2}\phi_{3,2}d_{i}^{2}-\frac{1}{2}\phi_{3,1}d_{i}^{2}\right)t+\\ \frac{1}{2}\left(\phi_{2,2}-\phi_{2,1}+\phi_{3,1}d_{i}+\phi_{3,2}d_{i}\right)t^{2}+\frac{1}{6}\left(\phi_{3,2}-\phi_{3,1}\right)t^{3}]\}. \end{array}$$

Equation (18) and the PSSAF in [11] have similar forms. Thus, Equation (18) follows the characteristic of PCFCRD [11]. If Equation (23) takes the LFM signal model, the cross-terms will form spurious peaks, which will influence the detection of the auto-terms. Equation (23) takes the LFM form when $\phi_{3,1}=\phi_{3,2}$. Let $\Delta \phi_{1}=\phi_{1,1}-\phi_{1,2}$, $\Delta \phi_{2}=\phi_{2,1}-\phi_{2,2}$, $\Delta \phi_{3}=\phi_{3,1}-\phi_{3,2}$. Through the analysis of Equation (23), two cases will be proposed to explain the cross-terms influences. They are listed as follows:

- $\Delta \phi_{1}\neq 0$, $\Delta \phi_{2}\neq 0$, $\Delta \phi_{3}=0$,
- $\Delta \phi_{1}\neq 0$, $\Delta \phi_{2}=0$, $\Delta \phi_{3}=0$.

In these two cases, the spurious peaks will appear on CR-QCR domains for the MPCRD. However, 2D-PMPCRD can greatly suppress the spurious peaks by introducing the multi-scale factor PASSF and mNUFFT-FFT. We will explain how the proposed algorithm suppresses the spurious peaks as follows.

#### 3.1.1. Case One

When $\Delta \phi_{1}\neq 0$, $\Delta \phi_{2}\neq 0$, $\Delta \phi_{3}=0$, the $R_{cross}\left(t,\tau ,d_{i}\right)$ can be presented as follows:

(24) $$\begin{array}{c} R_{cross}\left(t,d_{i}\right)=A_{1}A_{2}\exp\left\{j2\pi \left[\phi_{1,1}d_{i}+\phi_{1,2}d_{i}+\frac{1}{2}\phi_{2,1}d_{i}^{2}-\frac{1}{2}\phi_{2,2}d_{i}^{2}+\frac{1}{6}\phi_{3,1}d_{i}^{3}+\frac{1}{6}\phi_{3,2}d_{i}^{3}\right]\right\}\times \\ \exp\{j2\pi \left[\left(\phi_{1,1}-\phi_{1,2}+\phi_{2,1}d_{i}+\phi_{2,2}d_{i}\right)t+\frac{1}{2}\left(\phi_{2,1}-\phi_{2,2}+\phi_{3,1}d_{i}+\phi_{3,2}d_{i}\right)t^{2}\right]\}+\\ A_{1}A_{2}\exp\left\{j2\pi \left[\phi_{1,1}d_{i}+\phi_{1,2}d_{i}+\frac{1}{2}\phi_{2,2}d_{i}^{2}-\frac{1}{2}\phi_{2,1}d_{i}^{2}+\frac{1}{6}\phi_{3,1}d_{i}^{3}+\frac{1}{6}\phi_{3,2}d_{i}^{3}\right]\right\}\times \\ \exp\{j2\pi \left[\left(\phi_{1,2}-\phi_{1,1}+\phi_{2,1}d_{i}+\phi_{2,2}d_{i}\right)t+\frac{1}{2}\left(\phi_{2,2}-\phi_{2,1}+\phi_{3,1}d_{i}+\phi_{3,2}d_{i}\right)t^{2}\right]\}. \end{array}$$

From Equation (24), we can see that the $R_{cross}$ takes two LFM signals. Therefore, the cross-terms will form two spurious peaks. The spurious peaks coordinates are presented as

(25) $$\begin{array}{c} r_{i}^{cross1}=2\left(\phi_{1,1}-\phi_{1,2}+\phi_{2,1}d_{i}+\phi_{2,2}d_{i}\right),\omega_{i}^{cross1}=\phi_{2,1}-\phi_{2,2}+\phi_{3,1}d_{i}+\phi_{3,2}d_{i}, \end{array}$$

(26) $$\begin{array}{c} r_{i}^{cross2}=2\left(\phi_{1,2}-\phi_{1,1}+\phi_{2,1}d_{i}+\phi_{2,2}d_{i}\right),\omega_{i}^{cross2}=\phi_{2,2}-\phi_{2,1}+\phi_{3,1}d_{i}+\phi_{3,2}d_{i}. \end{array}$$

From Equation (22), it is easily seen that the auto-terms take two LFM signals form, which means that the auto-terms will form two true peaks. The coordinates of true peaks are presented as

(27) $$\begin{array}{c} r_{i}^{auto1}=4\phi_{2,1}d_{i},\omega_{i}^{auto1}=2\phi_{3,1}d_{i}, \end{array}$$

(28) $$\begin{array}{c} r_{i}^{auto2}=4\phi_{2,2}d_{i},\omega_{i}^{auto2}=2\phi_{3,2}d_{i}. \end{array}$$

From the analysis of the coordinates of the true peaks and the spurious peaks, we can find that the coordinates of true peaks are proportional to the scale factors. It can be presented as

(29) $$\begin{array}{c} P_{1,2}=\frac{\omega_{1}^{auto1}}{\omega_{2}^{auto1}}=\frac{r_{1}^{auto1}}{r_{2}^{auto1}}=\frac{\omega_{1}^{auto2}}{\omega_{2}^{auto2}}=\frac{r_{1}^{auto2}}{r_{2}^{auto2}}. \end{array}$$

However, the spurious peaks coordinates do not satisfy Equation (29). From Section 2.2, we can see that mNUFFT-FFT can be used to transform the auto-terms into the same position and the cross-terms into different positions on the CR-QCR domains. After performing the mNUFFT-FFT, we can enhance the true peaks and suppress the spurious peaks by multiplying all the CR-QCR domains. In order to prove the cross-term suppression performance, we consider the noiseless and noisy condition.

**Figure 1:**
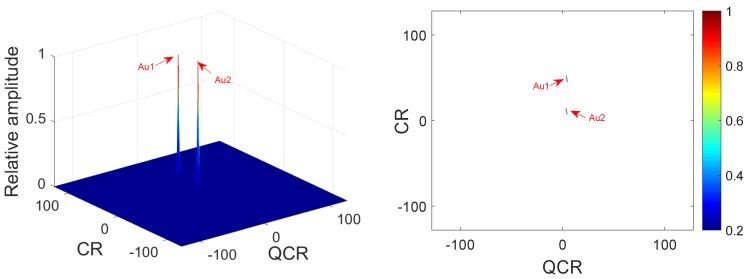
Cross-terms suppression of 2D-PMPCRD.

**Figure 2:**
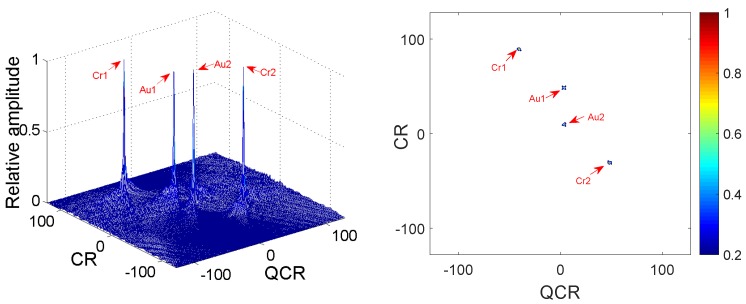
Cross-terms suppression of MLVD.

**Example** **1.** *Two QFM signals $P_{1}$, $P_{2}$ are considered to show the good cross-term suppression performance of the 2D-PMPCRD compared with MLVD in this example. The sampling frequency fs is set to 256 Hz. The signal length N and effective signal length $N_{e}$ are set as 624 and 512. The effective signal length $N_{e}$ is the length of the signal after the autocorrelation. Signal parameters are set as follows: $A_{1}=1$, $\phi_{1,1}=10$, $\phi_{2,1}=10$, $\phi_{3,1}=4$ for $P_{1}$; $A_{2}=1$, $\phi_{1,2}=40$, $\phi_{2,2}=50$, $\phi_{3,2}=4$ for $P_{2}$. The constant delay is set as 2 s. In this example, two scale factors are set as $d_{1}=56$, $d_{2}=64$ for 2D-PMPCRD, which are selected by parameter selection criterion and will be discussed in Section 3.2. Figure 1 gives simulation results of 2D-PMPCRD. Figure 2 gives the simulation results of MLVD with the scale factor equal to $d_{1}=56$.*

In Figure 1 and Figure 2 , Cr1, Cr2 and Au1, Au2 denote the spurious peaks and the true peaks. The CR and QCR axis denote the chirp rate and quadratic chirp rate of QFM signals. From Figure 2, we can see that there are four peaks on the CR-QCR domain by using MLVD. It is not hard to see that the spurious peaks are high enough to influence the detection of the true peaks. It is a great challenge for MLVD. Thus, the MLVD can not be used to estimate parameters of the QFM signals when multi-QFM signals have the same QCR. The 2D-PMPCRD overcomes the identifiability problem. From Figure 1, we can see that the true peaks are strengthened and the spurious peaks are greatly weakened after the 2D-PMPCRD operator. The simulation results have proven the theoretical analysis.

**Figure 3:**
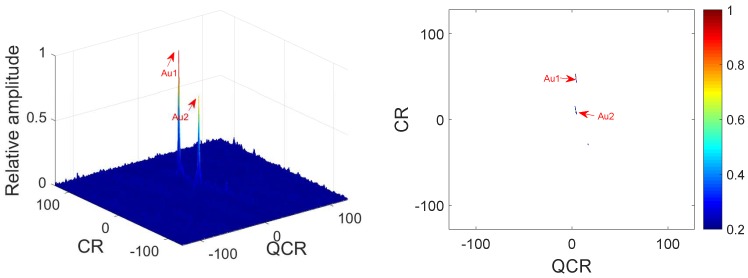
Cross-terms suppression of 2D-PMPCRD (SNR = 0 dB).

**Figure 4:**
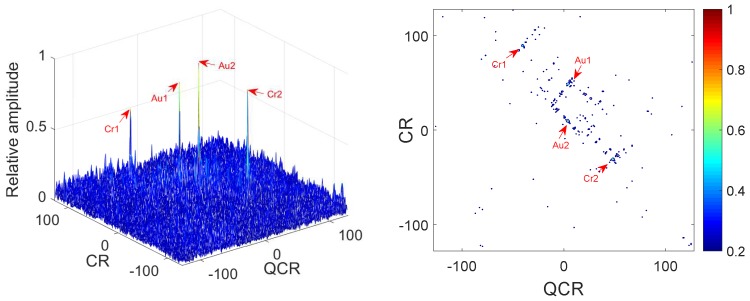
Cross-terms suppression of MLVD (SNR = 0 dB).

**Example** **2.** *In this example, we analyze the cross-terms suppression under noisy multi-QFM signals. The parameters of QFM signals are set the same as Example 1. The input SNR is set as 0 dB. Figure 3 gives simulation results of 2D-PMPCRD. Figure 4 gives the simulation results of MLVD.*

In Figure 3 and Figure 4, we can see that the true peaks are much higher than spurious peaks for the 2D-PMPCRD under noisy condition. However, the spurious peaks for the MLVD are high enough to influence the detection of the true peaks. Thus, the 2D-PMPCRD has better cross-term suppression ability than MLVD.

#### 3.1.2. Case Two

When $\Delta \phi_{1}\neq 0$, $\Delta \phi_{2}=0$, $\Delta \phi_{3}=0$, the $R_{cross}\left(t,\tau ,d_{i}\right)$ can be presented as follows:

(30) $$\begin{array}{c} R_{cross}\left(t,d_{i}\right)=A_{1}A_{2}\exp\left\{j2\pi \left[\phi_{1,1}d_{i}+\phi_{1,2}d_{i}+\frac{1}{6}\phi_{3,1}d_{i}^{3}+\frac{1}{6}\phi_{3,2}d_{i}^{3}\right]\right\}\times \\ \exp\{j2\pi \left[\left(\phi_{1,1}-\phi_{1,2}+\phi_{2,1}d_{i}+\phi_{2,2}d_{i}\right)t+\frac{1}{2}\left(\phi_{3,1}d_{i}+\phi_{3,2}d_{i}\right)t^{2}\right]\}+\\ A_{1}A_{2}\exp\left\{j2\pi \left[\phi_{1,1}d_{i}+\phi_{1,2}d_{i}+\frac{1}{6}\phi_{3,1}d_{i}^{3}+\frac{1}{6}\phi_{3,2}d_{i}^{3}\right]\right\}\times \\ \exp\{j2\pi \left[\left(\phi_{1,2}-\phi_{1,1}+\phi_{2,1}d_{i}+\phi_{2,2}d_{i}\right)t+\frac{1}{2}\left(\phi_{3,1}d_{i}+\phi_{3,2}d_{i}\right)t^{2}\right]\}. \end{array}$$

It is not hard to see that the coordinates of spurious peaks can be described as follows:

(31) $$\begin{array}{c} r_{i}^{cross1}=2\left(\phi_{1,2}-\phi_{1,1}+\phi_{2,1}d_{i}+\phi_{2,2}d_{i}\right),\omega_{i}^{cross1}=\phi_{3,1}d_{i}+\phi_{3,2}d_{i}, \end{array}$$

(32) $$\begin{array}{c} r_{i}^{cross2}=2\left(\phi_{1,1}-\phi_{1,2}+\phi_{2,1}d_{i}+\phi_{2,2}d_{i}\right),\omega_{i}^{cross2}=\phi_{3,1}d_{i}+\phi_{3,2}d_{i}. \end{array}$$

Because $\phi_{3,1}$ is equal to $\phi_{3,2}$ and $\phi_{2,1}$ is equal to $\phi_{2,2}$, we see that the auto-term will form a higher peak, which is different from case one. The position of the true peak is ($4\phi_{2:}d_{i},2\phi_{3:}d_{i}$), where $\phi_{2:}=\phi_{2,1}=\phi_{2,2}$ and $\phi_{3:}=\phi_{3,1}=\phi_{3,2},$ and it meets Equation (29). However, the coordinates of the spurious peaks do not meet Equation (29). Thus, the process to enhance the true peak and suppress the spurious peaks are the same as case one. In this case, we also consider the noiseless and noisy condition.

**Figure 5:**
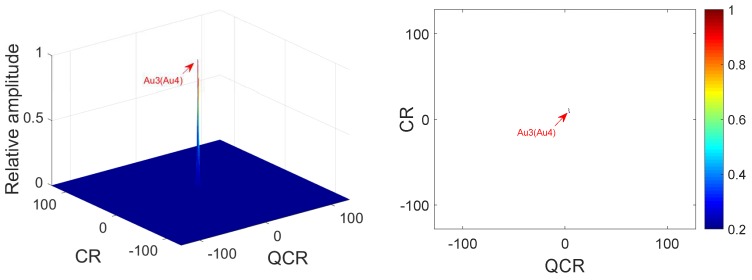
Cross-terms suppression of 2D-PMPCRD.

**Figure 6:**
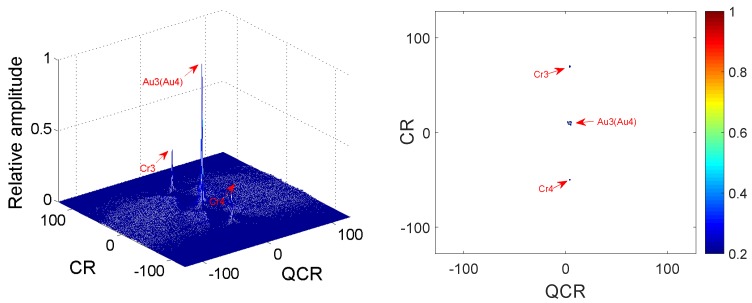
Cross-terms suppression of MLVD.

**Example** **3.** *Two QFM signals $P_{3}$, $P_{4}$ are considered to show the good cross-term suppression performance of the 2D-PMPCRD compared with MLVD in this example. The sampling frequency fs is set as 256 Hz. The signal length N and effective signal length $N_{e}$ are set as 624 and 512. Signal parameters are set as follows: $A_{3}=1$, $\phi_{1,3}=10$, $\phi_{2,3}=10$, $\phi_{3,3}=4$ for P3; $A_{4}=1$, $\phi_{1,4}=40$, $\phi_{2,4}=10$, $\phi_{3,4}=4$ for P4. The constant delay is set as 2 s. In this example, two scale factors, which are $d_{1}=56$ and $d_{2}=64$, are selected for 2D-PMPCRD. Figure 5 gives simulation results of 2D-PMPCRD. Figure 6 gives the simulation results of MLVD with the scale factor equal to $d_{1}=56$.*

In Figure 5 and Figure 6, Cr3, Cr4 and Au3, Au4 denote the spurious peaks and the true peaks. From Figure 6, we can see that two spurious peaks appear on the CR-QCR domain, which will influence the detection of the true peak. Thus, MLVD can not be used under this condition. From the analysis of Equation (22), we can see that the true peaks will appear on the same position on the CR-QCR domain when the multi-QFM signals have the same QCR and CR. From Figure 6, we can see that the simulation results have proven the theoretical analysis.

**Figure 7:**
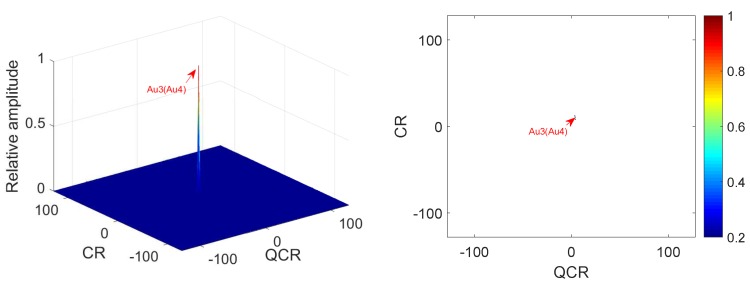
Cross-terms suppression of 2D-PMPCRD (SNR = 0 dB).

**Figure 8:**
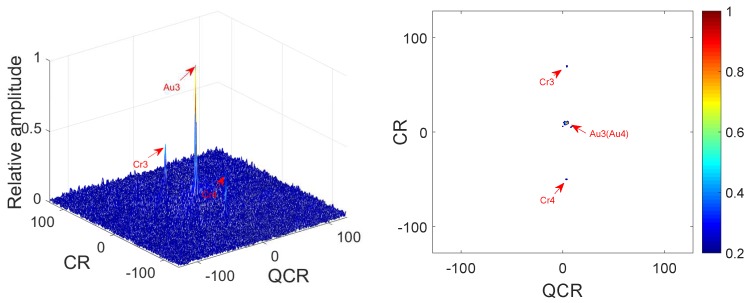
Cross-terms suppression of MLVD (SNR = 0 dB).

**Example** **4.** *In this example, we analyze the cross-terms suppression under noisy multi-QFM signals. The parameters of QFM signals are set the same as Example 3. The input SNR is set as 0 dB. Figure 7 gives simulation results of 2D-PMPCRD. Figure 8 gives the simulation results of MLVD.*

In Figure 7 and Figure 8, we can see that the simulation results are the same as Example 3. From Figure 5 and Figure 7, we can see that the difference between Figure 5 and Figure 7 is not obvious. In fact, there are differences between Figure 5 and Figure 7 if we calculate the peak to mean background ratio (PMR), which is the ratio of the max value of the modulus of $Q_{2D-PMPCRD}\left(r,w,D\right)$ to the mean value of the modulus of *Q*${}_{2D-PMPCRD}$(r,w,D). The PMRs of Figure 5 and Figure 7 are about 42.07 dB and 33.24 dB, respectively. We can see that the 2D-PMPCRD also has good cross-term suppression performance under noisy environment. From the above simulation analysis, it is easy to see that the 2D-PMPCRD has better cross-term suppression ability than MLVD and can solve the identifiability problems of MLVD.

### 3.2. Parameter Selection Criterion

In this section, the selection criterion of the constant delay *h*, the scale factor $d_{i}$ and the number of scale factors *L* are analyzed.

#### 3.2.1. Selection of Constant Delay

According to the analysis in [10,11], we know that the constant delay *h* is related to signal length and anti-noise performance. It can benefit the anti-noise ability if we set a suitable value. Thus, in this section, we discuss the select of the constant delay *h*.

With the analysis in [11], we can find that the anti-noise ability achieves its best when $h\geq N_{e}*T_{s}$. Since the effective length $N_{e}$ of the signal is finite in realistic application, *h* should not be increased without limit. Here, considering the anti-noise performance and the effective length of the signal, the constant delay *h* is set as

(33) $$\begin{array}{c} h=N_{e}*T_{s}. \end{array}$$

In realistic applications, we have much data that are stored in the storage. Therefore, we can use them to satisfy the constant delay [10].

#### 3.2.2. Selection of the Scales Factors and Number of Scale Factors

Through the analysis in [21], we can see that the scale factors can not only reduce the signal order, but also benefit the anti-noise performance. The selection criteria of scale factor $d_{i}$ is to obtain the smallest MSE of QCR and CR according to [15]. Through the analysis in [15], the optimal $d_{i}$, which is denoted as $d^{opt},$ can be presented as

(34) $$\begin{array}{c} d^{opt}\approx \left\lceil 0.089N\right\rceil , \end{array}$$

where ⌈∗⌉ denotes the round up function. *N* denotes the signal length.

According to [17], we can see that the optimal choice of *L* lags are $d_{1}=d_{2}=\cdots =d_{L-1}=d^{opt}$. Thus, the optimal scale factors of 2D-PMPCRD are $d_{1}=d_{2}=\cdots =d_{L-1}=\left\lceil 0.089N\right\rceil$. However, in Section 3.1, we can see that the cross-terms form spurious peaks if the scale factors are set as the same. The spurious peaks influence the detection of the true peaks. To solve this problem, the simple method is to use different scale factors. Thus, the scale factors are usually selected as different ones in realistic application. Considering the accuracy of CR and QCR estimation, the sub-optimal scale factors should be selected around the optimal one [17].

From the above analysis, we can see that the selection of the number of scale factors *L* is also very important for the performance of 2D-PMPCRD. According to [17], estimation performance will be improved as the number of scale factors *L* increases. However, we can see that the anti-noise performance is improved slightly when L≥2. In this paper, from Section 3.1, we can see that the proposed algorithm can acquire good cross-term suppression performance when *L* is set to 2. Furthermore, from Section 2, we can see that the computation cost will increase rapidly as the number of scale factors *L* increases. Thus, considering the computation cost and performance, *L* is usually selected as 2.

## 4. Anti-Noise Performance and Computation Cost Analysis

In this section, anti-noise performance and computation will be analyzed for 2D-PMPCRD. Both the mono-component QFM signals and multi-QFM signals are considered to prove the good anti-noise performance of 2D-PMPCRD.

### 4.1. Anti-Noise Performance Analysis

In this section, we propose Examples 5 and 6 to illustrate the anti-noise performance of 2D-PMPCRD.

**Example** **5.** *One QFM signal that denotes $P_{5}$ is considered. The sampling frequency fs is set as 256 Hz. The signal length N and effective signal length $N_{e}$ are set as 624 and 512. Parameters for $P_{5}$ are set as follows: $A_{5}=1$, $\phi_{1,5}=10$, $\phi_{2,5}=10$, $\phi_{3,5}=4$. The set of scale factors are set to $d_{1}=56$, $d_{2}=64$. The constant delay h is set to 2 s. The test input SNRs in figures are SNR = [−8:1:−2]. In this example, 100 simulations are run for each SNR value. The input–output SNR [21] is used as a measurement and the result is shown in Figure 5a. The observed mean square error (MSE) and the Cramer–Rao Lower Bound (CRLB) of quadratic chirp rate and chirp rate in different SNRs are shown in Figure 5b,c, respectively.*

**Figure 9:**
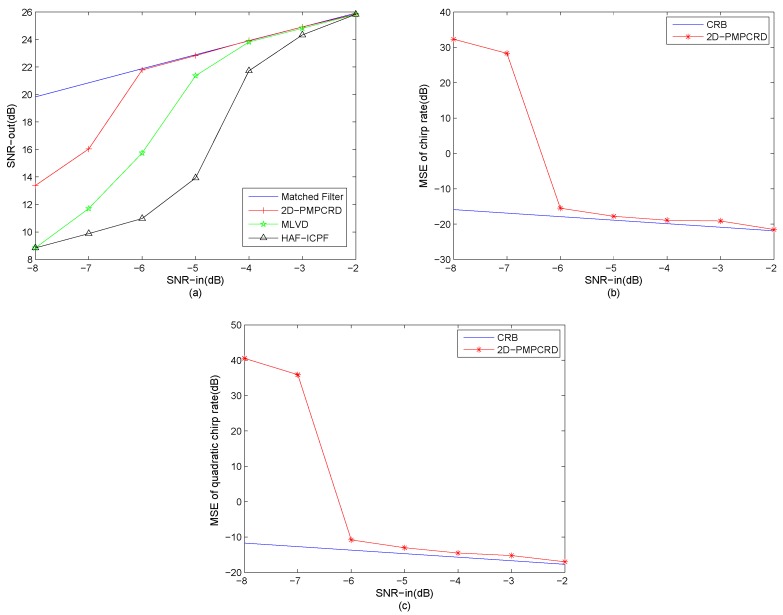
Anti-noise performance analysis for mono-QFM signal. (**a**) Input–output SNR; (**b**) MSEs of the CR estimation; (**c**) MSEs of the QCR estimation.

From Figure 9, we can see that the anti-noise performance of HAF-ICPF is the worst. The anti-noise performance of MLVD is better than the HAF-ICPF. However, the threshold SNR of MLVD is still high. The anti-noise performance of 2D-PMPCRD is the best.

The HAF-ICPF uses HAF to reduce the coefficient order of QFM signals. However, HAF is sensitive to noise and suffers from the error propagation. Thus, from Figure 9a, we can see that the threshold SNR of HAF-ICPF is only −2 dB. The evidence of the simulation results can also be found in [15,20]. The MLVD introduces a novel PSSAF and the keystone transform to transform the QFM signals into CR-QCR domain. The MLVD can not only improve the anti-noise performance, but also overcome the error propagation by estimating the CR and QCR together on the CR-QCR domain. However, the MLVD only uses one scale factor, which is not suitable in low SNR. From Figure 9a, we can see that the threshold SNR of MLVD is −4 dB. The evidence of the simulation result can also be found in [21]. The 2D-PMPCRD uses a constant delay *h* and multi-scale factors D to improve the performance in low SNR. In addition, the multiplying operation of different CR-QCR domains can also improve the anti-noise performance. Therefore, the anti-noise performance of 2D-PMPCRD is the best. The threshold SNR of 2D-PMPCRD is −6 dB.

In Figure 9b,c, the MSEs of CR and QCR estimations are plotted as functions of SNR. From the Figure 9b,c, we can see that, for the 2D-PMPCRD, the MSEs of CR and QCR estimations increase abruptly at −6 dB. This is because, if the SNR is lower than the threshold SNR, there is high probability that the maximum peak on the CR-QCR domain are some random peaks, unrelated to ($\phi_{2,5},\phi_{3,5}$) of the QFM signal. This will cause the parameter estimation algorithm to fail. Therefore, the MSEs of CR and QCR increase abruptly at the threshold SNR.

**Figure 10:**
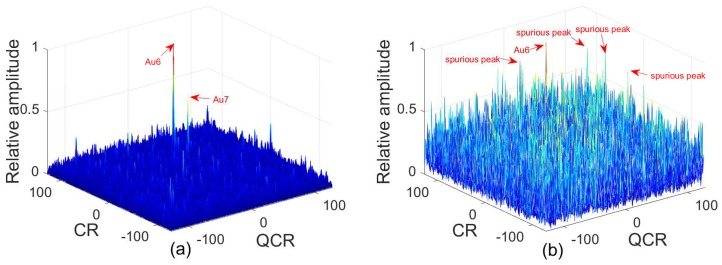
Anti-noise performance analysis for multi-QFM signals (SNR = −3 dB). (**a**) Anti-noise performance of 2D-PMPCRD; (**b**) anti-noise performance of MLVD.

**Example** **6.** *Two QFM signals $P_{6}$, $P_{7}$ are considered to show the good anti-noise performance of the 2D-PMPCRD compared with MLVD under noisy multi-QFM signals. The sampling frequency fs is set as 256 Hz. The signal length N and the effective signal length $N_{e}$ are set as 624 and 512. Signal parameters are set as follows: $A_{6}=1$, $\phi_{1,6}=10$, $\phi_{2,6}=10$, $\phi_{3,6}=4$ for $P_{6}$; $A_{6}=1$, $\phi_{1,7}=40$, $\phi_{2,7}=50$, $\phi_{3,7}=12$ for $P_{7}$. The constant delay is set as 2 s. In this example, two scale factors, which are $d_{1}=56$ and $d_{2}=64$, are selected for 2D-PMPCRD. SNR is set as −3 dB. Figure 10a gives simulation results of 2D-PMPCRD. Figure 10b gives the simulation results of MLVD with the scale factor equal to $d_{1}=56$.*

From Figure 10, we can see that 2D-PMPCRD can distinguish these two QFM signals. The true peaks of MLVD are immersed in the noise. Therefore, 2D-PMPCRD has better anti-noise performance than MLVD when noisy multi-QFM signals are considered.

### 4.2. Computation Cost Analysis

Computation cost is an important factor that should be considered in realistic application. In this section, the computation cost of 2D-PMPCRD will be discussed and the computation cost of the comparison algorithms HAF-ICPF and MLVD will also be analyzed.

For HAF-ICPF, the main procedures are HAF and ICPF, and their computation costs are (O(N)) [14] and $\left(O\left(N^{3}\right)\right)$ [30,31], respectively. Thus, the computation cost of HAF-ICPF is $\left(O\left(N^{3}\right)\right)$. For MLVD, the main procedures are PSSAF and keystone transform. The computation of PSSAF is $\left(O\left(N^{2}\right)\right)$ [21]. The keystone transform can be divided into FFT and IFFT [21]. Thus, the computation cost of keystone transform is similar to FFT. As we all know, the computation of FFT is $\left(O\left(N^{2}log_{2}N\right)\right)$. Thus, the computation cost of MLVD is $\left(O\left(N^{2}log_{2}N\right)\right)$ [21]. The main procedures of 2D-PMPCRD are PSSAF, dechirp operation, mNUFFT-FFT and L−1 products of MPCRD. The computation cost of the PSSAF of 2D-PMPCRD is similar to MLVD, thus its computation cost is $\left(O\left(N^{2}\right)\right)$. The computation cost of dechirp operation is $\left(O\left(N^{2}\right)\right)$ [11]. From the analysis of Section 3.1, we know that the mNUFFT-FFT can be divided into NUFFT and FFT. Thus, the computation cost of the mNUFFT-FFT is the same as NUFFT and FFT, whose computation costs are $\left(O\left(N^{2}log_{2}N\right)\right)$ [11,28,29]. In realistic application, *L* is much smaller than the signal effective length (e.g., 2). Thus, the computation of 2D-PMPCRD is $\left(O\left(N^{2}log_{2}N\right)\right)$. The computation costs of these three algorithms are listed in Table 1.

From Table 1, we can see that the computational cost of HAF-ICPF is the worst. The computational cost of 2D-PMPCRD is equivalent to MLVD. Therefore, 2D-PMPCRD can obtain better cross-terms suppression performance and anti-noise performance with reasonable computational cost. Compared with HAF-ICPF and MLVD, the proposed algorithm is more suitable for the real-time ISAR imaging system.

## 5. Conclusions

In this paper, we propose the 2D-PMPCRD to estimate CR and QCR of the QFM signals by introducing a novel multi-scale PSSAF and the mNUFFT-FFT. The PSSAF utilizes the multi-scale factors and the constant delay to improve the anti-noise performance. The mNUFFT-FFT transforms the auto-terms into the same position and the cross-terms into different positions on CR-QCR domains. By multiplying all the CR-QCR domains, 2D-PMPCRD can greatly weaken the cross-terms while strengthening the auto-terms. Meanwhile, the operation of multiplying can also improve the anti-noise performance. Furthermore, the 2D-PMPCRD can be easily implemented by using the multi-scale PSSAF, the mNUFFT-FFT and the complex multiplication. The simulation results verify that the 2D-PMPCRD has obvious superiorities in cross-term suppression and improves the identifiability ability under multi-QFM signals’ situation. The 2D-PMPCRD can greatly improve the anti-noise performance with reasonable computation cost, and its threshold SNR reaches −6 dB.

## Figures and Tables

**Table 1:** Computation cost of algorithms.

Estimation Algorithm	Computation Cost
HAF-ICPF	$O(N^{3})$
MLVD	$O(N^{2}log_{2}N)$
2D-PMPCRD	$O(N^{2}log_{2}N)$
